# Response to R-CHOP in HPV-related squamous cell carcinoma of base of tongue: a case report

**DOI:** 10.1186/s41199-018-0028-6

**Published:** 2018-05-24

**Authors:** Ting Martin Ma, Hyunseok Kang, Steven P. Rowe, Ana P. Kiess

**Affiliations:** 1 Department of Radiation Oncology and Molecular Radiation Sciences, The Johns Hopkins University School of Medicine, Baltimore, MD 21231 USA; 2 Department of Oncology, The Johns Hopkins University School of Medicine, Baltimore, MD 21287 USA; 3 The Russell H. Morgan Department of Radiology and Radiological Science, The Johns Hopkins University School of Medicine, Baltimore, MD 21287 USA

**Keywords:** Squamous cell carcinoma, Non-Hodgkin’s lymphoma, R-CHOP, Synchronous, HPV

## Abstract

**Background:**

Synchronous squamous cell carcinoma of the head and neck (HNSCC) and non-Hodgkin’s lymphoma is a rare clinical scenario. It is unknown whether the R-CHOP chemotherapy for lymphoma would also be active against HNSCC. Herein, we present such a case and a review of the literature.

**Case presentation:**

A 64 year-old female presented with painless jaundice. CT demonstrated a retroperitoneal mass and pathology showed follicular lymphoma. A base-of-tongue HPV^+^ squamous cell carcinoma was found incidentally on staging CT. R-CHOP chemotherapy was initiated. After 3 cycles of R-CHOP the lymphoma had a complete metabolic response and, unexpectedly, the HNSCC also demonstrated excellent response. The patient received another 3 cycles followed by radiation to the HNSCC and to date is in remission for both cancers.

**Conclusions:**

This case highlights the exquisite sensitivity of HPV-related HNSCC, which should be taken into consideration in treatment prioritization of a concurrent diagnosis of a second cancer.

## Background

Squamous cell carcinoma (SCC) accounts for more than 90% of tumors in the head and neck [1]. For patients with head and neck squamous cell carcinoma (HNSCC), a synchronous second primary cancer (SPC) has been reported in 1–5% of cases [2, 3]. Typically SPCs are also SCCs. An SPC of lymphogenic origin is extremely rare. In one study, 3.5% of the SPCs were non-Hodgkin lymphoma (NHL) with a majority of the index primaries seen in the oropharynx (39.2%) [3]. With the emergence of human papillomavirus (HPV) as a distinct risk factor for oropharyngeal HNSCC, the risk of SPC carried by oropharyngeal cancers has decreased [2]. On the other hand, there is a growing body of evidence demonstrating that patients with NHL or chronic lymphoid leukemia are immunosuppressed, partially attributed to disease biology itself, and are more susceptible to other malignancies including cutaneous SCC [4–10]. Various mechanisms of immune escape in NHL have been described, including impaired HLA-mediated cancer cell recognition [11], deranged apoptotic mechanisms, and changes in the tumor microenvironment involving regulatory T cells and tumor-associated macrophages [12–15].

Here, we report a unique case of synchronous follicular lymphoma and HPV^+^ squamous cell carcinoma at base of tongue in which the SCC demonstrated an excellent response after only 3 cycles of R-CHOP chemotherapy. We also review the literature and cite other cases of synchronous SCC of aerodigestive tract and lymphoma treated with upfront R-CHOP chemotherapy, with a discussion of possible mechanisms of how component(s) of R-CHOP chemotherapy led to the regression of SCC.

## Case presentation

**Fig. 1:**
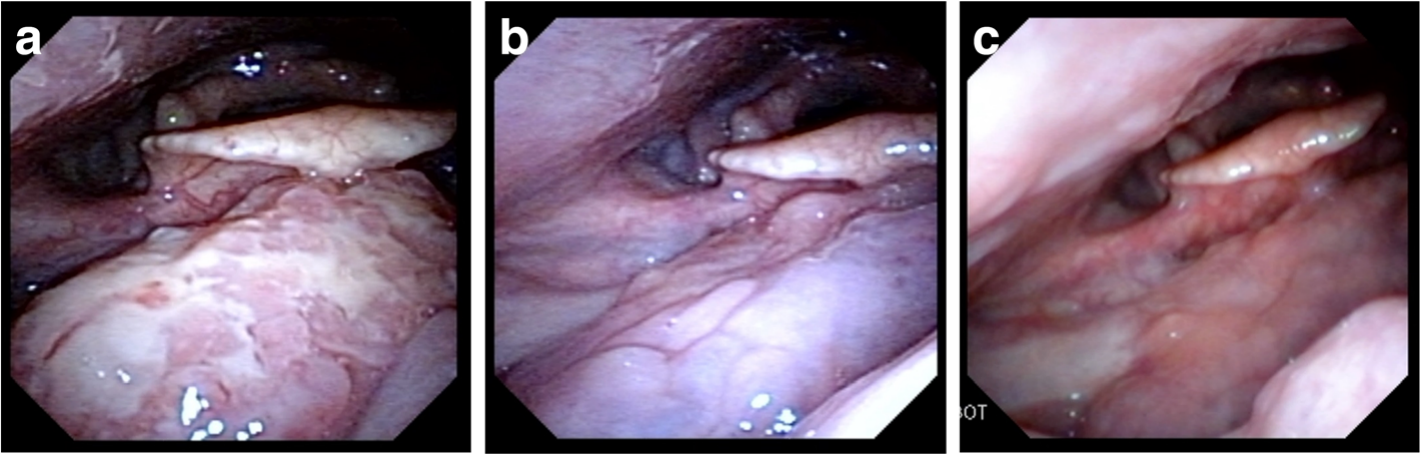
Flexible nasopharyngolaryngoscopy view of the right BOT mass before treatment (**a**), after 3 cycles of R-CHOP chemotherapy (**b**) and after the completion of 6 cycles of R-CHOP chemotherapy (**c**)

**Fig. 2:**
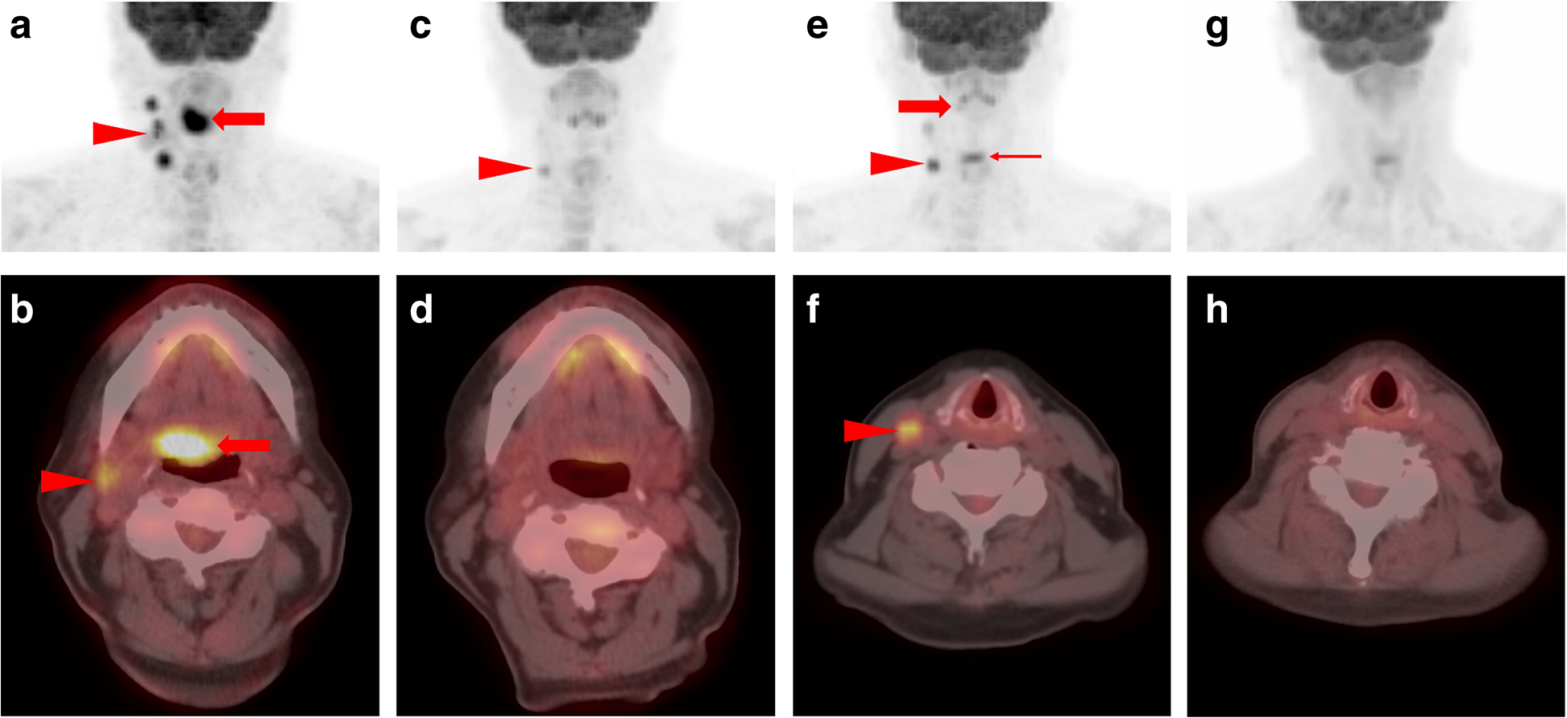
**a** Baseline head and neck maximum intensity projection (MIP) image demonstrating focal FDG uptake in the patient’s BOT HNSCC (red arrow) as well as ipsilateral cervical adenopathy (red arrowhead). **b** Representative axial PET/CT slice from the same time point as in (**a**) which delineates the BOT HNSCC (red arrow) and also highlights one of the right-sided cervical lymph nodes (red arrowhead). **c** Head and neck MIP image following 3 cycles of R-CHOP demonstrates complete metabolic response in the patient’s BOT HNSCC and partial response in the ipsilateral cervical adenopathy (red arrowhead). **d** Axial PET/CT image from the same time point as (**c**) shows no abnormal uptake at the BOT (persistently FDG-avid cervical nodes are not shown on this slice). **e** Head and neck MIP image following completion of R-CHOP therapy demonstrates very subtle increased uptake in the BOT HNSCC (red arrow, barely visible) and increasing uptake in ipsilateral cervical lymph nodes (red arrowhead). Note normal physiologic activity in the vocal cords (thin red arrow). **f** Representative axial PET/CT image through the neck shows an FDG-avid right level III lymph node compatible with residual HNSCC. **g** Head and neck MIP and (**h**) axial PET/CT images following completion of chemoradiation therapy show no evidence of metabolically active primary or nodal HNSCC

**Fig. 3:**
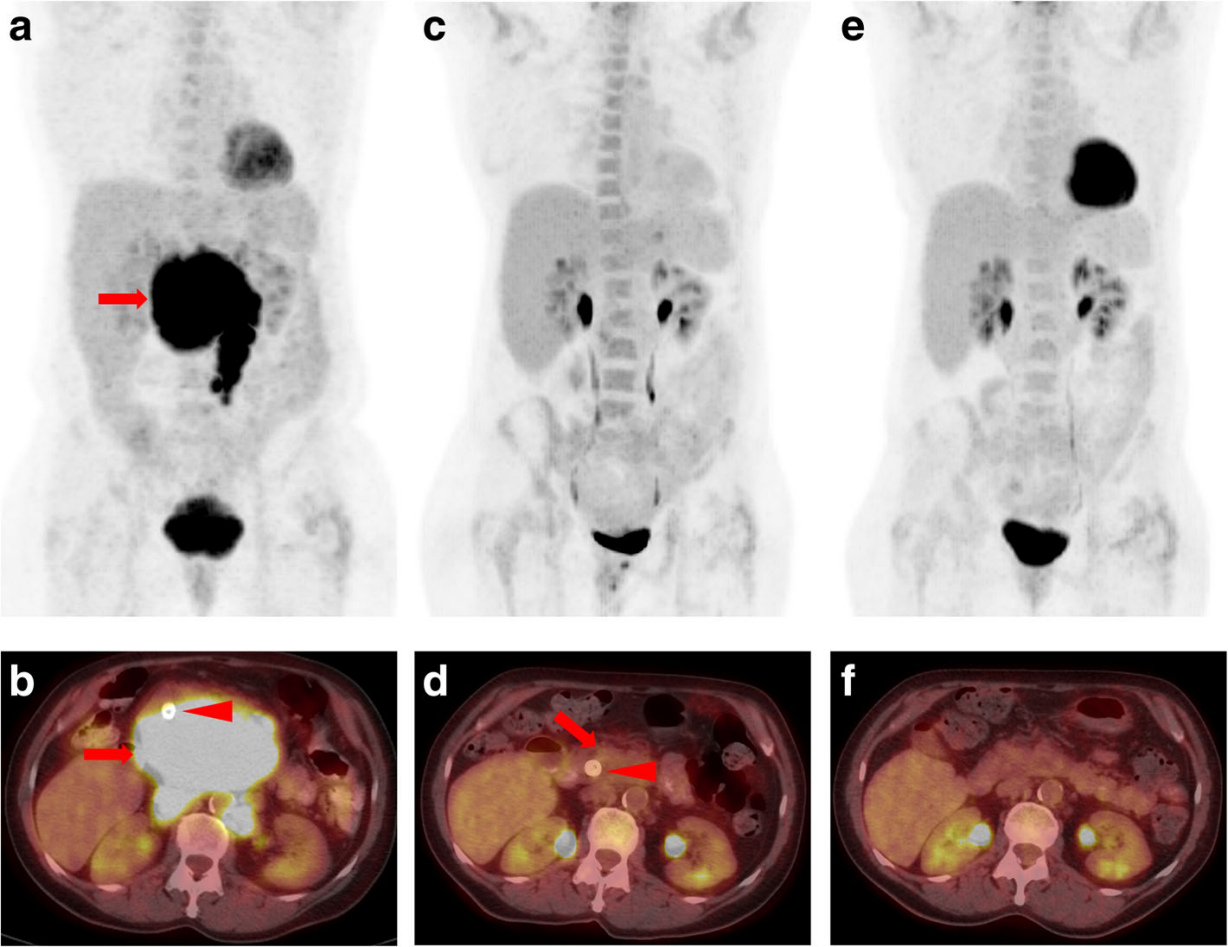
**a** Baseline whole-body MIP image demonstrating intense FDG uptake in a large retroperitoneal mass (red arrow) compatible with patient’s follicular lymphoma. **b** Representative axial PET/CT image from the same time point as in (**a**) showing the large, FDG-avid mass (red arrow). Note the common bile duct stent (red arrowhead) that is markedly anteriorly displaced by the lymphomatous mass and explains the patient’s presentation with obstructive jaundice. **c** Whole-body MIP image following three cycles of R-CHOP shows no residual metabolically active lymphoma. **d** Representative axial PET/CT image from the same time point as in (**c**) is notable for the presence of minimal residual abnormal soft tissue in the retroperitoneum (red arrow, Lugano 2), with uptake equal to blood pool, compatible with a complete metabolic response. The common bile duct stent is in near-orthotopic location now that the retroperitoneal mass has dramatically reduced in size (red arrowhead). **e** Whole-body MIP image at the end of therapy, again demonstrating no metabolically active tumor. **f** Representative axial PET/CT image from the same time point as in (**e**) again depicts the complete metabolic response (Lugano 1) and also the removal of the common bile duct stent

A 64 year-old Caucasian female former smoker (4 pack-year) originally presented to the emergency department with painless jaundice. Physical exam revealed an afebrile female with scleral icterus and jaundice. Her abdomen was soft, non-tender, and non-distended in all quadrants with normal bowel sounds and no organomegaly. CT imaging demonstrated a large (10 cm) retroperitoneal mass, necessitating biliary stenting. Fine needle aspiration of the mass revealed a CD10^+^clonal B cell population by flow cytometry, consistent with presumptive B cell lymphoma. During the staging workup for the lymphoma, right-sided cervical level IIA and III lymphadenopathy was found incidentally during a routine dental check-up, which was initially thought to be of the same disease process. She had no supraclavicular or axillary lymphadenopathy. CT demonstrated right level II/III LN and possible right base of tongue (BOT) mass. Flexible laryngoscopy revealed an exophytic mass involving the right BOT that extended to the right glosso-tonsillar sulcus and beyond the midline measuring approximately 3 cm (Fig. 1). Excisional biopsy of two right cervical lymph nodes unexpectedly demonstrated squamous cell cancer (SCC) that was positive for p16 and HPV. Subsequently, positron emission tomography/computed tomography (PET/CT) demonstrated an FDG-avid right BOT mass (2.3 × 0.9 cm) with right-sided level IIA, IIB and III lymphadenopathy (all < 3 cm), consistent with biopsy-proven HPV-associated SCC (Fig. 2). There was also an intensely FDG-avid retroperitoneal mass (8.2 × 13.4 × 10.7 cm) along with left mesenteric, left periaortic, and left retroperitoneal lymph nodes (Fig. 3). Laparoscopic biopsy of gastroepiploic, mesenteric, and gastrocolic lymph nodes confirmed follicular lymphoma. Pathology showed relatively low number of centroblasts (fewer than 15 per high power field) compatible with low grade follicular lymphoma (WHO grade 1–2) with significantly elevated Ki-67 proliferation index (~ 80%) suggesting clinical behavior similar to WHO grade 3 follicular lymphoma. Omentum and liver were not involved. Therefore, a diagnosis of synchronous stage IV T2N2bM0 HPV^+^ SCC of right BOT and stage IIAX follicular lymphoma was made. At the time, she was relatively asymptomatic from the BOT cancer. She denied dysphagia, odynophagia, trismus, otalgia, or speech or voice change. She also denied night sweats, fevers, significant weight loss, or infectious symptoms. Videofluoroscopic swallow study evaluation was normal. ECOG performance status was 1. After stenting, the patient’s bilirubin normalized and she was asymptomatic. Her case was discussed at multidisciplinary case conferences, and the initial plan was to treat the BOT cancer first due to its likely curability and shorter treatment course.

One month later, however, the patient was admitted to the hospital because of worsening abdominal pain. Given concern for lymphoma becoming increasingly symptomatic, R-CHOP (rituximab, cyclophosphamide, doxorubicin, vincristine, and prednisone) chemotherapy was initiated. She tolerated therapy well and had resolution of abdominal pain. After completion of 3 cycles of R-CHOP, PET/CT scan demonstrated interval markedly decreased size and uptake of the retroperitoneal mass, as well as interval resolution of the FDG-avid BOT lesion, and most of the FDG-avid cervical lymph nodes (Figs. 2 and 3). She had no symptoms referable to lymphoma at this time. Nasopharyngolaryngoscopy also revealed no residual fullness in the area of the right BOT (Fig. 1). After completion of another 3 cycles of R-CHOP (in total 6 cycles), PET/CT scan demonstrated sustained metabolic resolution of the abdominal mass. However, FDG-avid right BOT lesion as well as right cervical level II and III nodes had become slightly more prominent compared to the end of cycle 3. The decision was made to start 7-week concurrent chemoradiation with weekly cisplatin 40 mg/m^2^ for the SCC. Unfortunately, she was found to be neutropenic and cisplatin was switched to cetuximab. At the end of the first cetuximab infusion, she developed a Grade 3 infusion reaction with rigors and chest pain and was diagnosed with NSTEMI. An attempt to re-initiate cisplatin treatment after ANC normalized was unsuccessful as patient experienced fever and altered mental status necessitating hospital admission. She had received a total of one dose of cetuximab and two doses of cisplatin before the decision was made to proceed with radiation therapy (RT) without further chemotherapy. In total, the patient received 6996 cGy, 212 cGy per day in 33 fractions with coverage of the oropharynx and bilateral neck using tomotherapy-based image-guided intensity-modulated radiation therapy. Despite experiencing significant anterior mouth sores from cetuximab early in treatment, as well as significant oropharynx mucositis late in treatment, she was ultimately able to complete RT without an enteral feeding tube. At 3-month follow-up, she had no clinical or radiographic evidence of disease on exam or PET/CT scan. At the time of this manuscript submission, 3 years after completion of the radiation therapy, she remained in remission for both cancers.

## Discussion

SCC is the most common malignant tumor of the head and neck and may arise in the oral cavity, pharynx, larynx, or sinonasal cavities. Although most HNSCCs are related to alcohol and/or tobacco use, the incidence of HPV-associated HNSCC is on the rise worldwide [16]. In the United States, more than half of cancers diagnosed in the oropharynx are linked to HPV type 16 [17]. Interestingly, one Danish study demonstrated that HPV infection is associated with an increased incidence of both Hodgkin and NHL using conization as a surrogate marker [18]. Therefore it is plausible that in this case, chronic immune activation induced by persistent HPV infection and the failure of the immune system to clear HPV infection and to control lymphoma development could have contributed to lymphomagenesis in addition to its role in the pathogenesis of HNSCC.

HPV^+^ OPC is a distinct type of OPC and has very different biology compared to its HPV^−^ counterparts. Patients with HPV-related OPCs have a more favorable prognosis, in part due to the natural biology of the cancer and in part because these tumors are more responsive to chemotherapy and radiotherapy than HPV^−^ cancers [19, 20]. Definitive treatment of locoregionally advanced (III/IV) OPC often requires a multimodality approach that may include chemotherapy, RT, concurrent chemoradiation (CRT) and/or surgery. Cisplatin is considered the gold standard for CRT, with cetuximab as an alternative agent [21]. Other common chemotherapy agents including paclitaxel, docetaxel, 5-FU, hydroxyurea and carboplatin have also been used in treating OPC.

An unexpected observation in this case was the excellent response of BOT HNSCC to R-CHOP chemotherapy intended for follicular lymphoma, even after only 3 cycles. Despite slight regrowth seen at the end of cycle 6, the  SCC responded very well to the initial 3 cycles of R-CHOP, judged by the interval resolution of the FDG-avid BOT lesion and most of the FDG-avid cervical lymph nodes and negative nasopharyngolaryngoscopy results. The patient was also free of symptoms from SCC. The observed effect of R-CHOP on HPV^+^ head and neck cancer was unexpected because components of this regimen do not overlap with any routine chemotherapy regimen for HNSCC. A literature search revealed 3 case reports of synchronous SCC of aerodigestive tract and lymphoma treated with upfront R-CHOP chemotherapy (Table 1). Lymphoma achieved complete response in 2 cases [22, 23] and partial response in the other [24]. In contrast, SCC achieved partial response in 1 case [22] and stability/progression in 2 cases [23, 24]. Of note, none of these cases were HPV-related SCC.

**Table 1:** Case reports of synchronous SCC of aerodigestive tract and lymphoma treated with upfront R-CHOP chemotherapy

Study	Patient characteristics	Index primary	Synchronous secondary primary	Treatment regimen	Response	Remarks
Morita et al., 2009 [22]	75-year-old female	DLBCL (lower lip)	SCC (buccal mucosa)	6 cycles of R-THP-COP	Complete response for DLBCL, partial response for SCC	SCC was subsequently treated with tegafur, gimeracil and oteracil potassium with partial response
Oikonomou et al., 2013 [24]	72-year-old male, 20 pack-yr smoking hx	BALT Lymphoma (LLL)	low-differentiated lung SCC (RML)	3 cycles of R-CHOP	Significant reduction in size of lymphoma and stability of the lung SCC	9-month follow-up CT revealed progression of the lung cancer with distant metastatic disease
Fujii et al., 2014 [23]	68-year-old female	DLBCL (Left cervical LNs)	Lung SCC (RUL and hilar and mediastinal LNs)	3 cycles of R-CHOP	Complete response for DLBCL; pulmonary SCC and right hilar LN stable/increased	Radical surgery performed after 3 cycles of R-CHOP to resect lung SCC

*DLBCL* diffuse large B-cell lymphoma, *SCC* squamous cell carcinoma, *BALT* bronchial-associated lymphoid tissue, *LLL* left lower lobe, *RML* right middle lobe, *RUL* right upper lobe, *LN* lymph node, *R-THP-COP* rituximab, pirarubicin, cyclophosphamide, vincristine, and prednisolone

Among the components of R-CHOP, only 2 agents have been evaluated as single chemotherapy agents in HNSCC. Cyclophosphamide had a response rate of 36% in 77 patients in one study [25] and doxorubicin had a response rate of 24% in another study [25]. Vinblastine, a closely related agent to vincristine, demonstrated a response rate of 29% [25]. However, these early studies should be interpreted with caution due to lower prevalence of HPV-related SCC during the study period [16], limited size, and a lack of information concerning prior treatment and nutritional and performance status.

Historically, these agents have also been evaluated as combination therapies with other chemotherapy agents, mostly in the 1980’s and 1990’s. Many cyclophosphamide combinations have been reported, as this is an agent with very broad activity in a variety of epithelial tumors. The most common combination utilized was with bleomycin, methotrexate, and 5-FU. The overall response rate was 47% (132/279) with a range of 11–69% [26].

Vincristine has been mostly reported as a combinatorial agent with cyclophosphamide, cisplatin, bleomycin, methotrexate, and 5-FU in HNSCC [26–28]. For example, the combination of vincristine, bleomycin, and methotrexate produced a response rate of the primary tumor in 61% [29]. As part of the CABO (cisplatin, methotrexate, bleomycin and vincristine) regimen, the overall response rate was 34% in a phase III trial of recurrent or metastatic HNSCC [30].

The role of B cells in solid tumors has also been under intense examination. B cells can exert their tumorigenic effects by secretion of paracrine factors that sustain chronic inflammation [31], deposition of immune complexes and Fcγ receptor-dependent activation of myeloid cells, and by enhancing T_H_2-type CD4^+^ T helper cells while repressing CD4^+^ T_H_1 cells which influence CD8^+^ cytotoxic T cell activity [32]. As human SCCs of the vulva and head and neck exhibit hallmarks of B cell infiltration, it is postulated that rituximab, a chimeric monoclonal antibody against CD20 that leads to B cell depletion [33], could be considered in solid tumors [32]. Indeed, in a preclinical murine model of HPV16-related SCC, administration of rituximab to mice bearing preexisting SCCs improved response to platinum- and taxol-based chemotherapy, although it was ineffective as a single agent. This process was dependent on expression of an altered repertoire of chemokines expressed by macrophages, resulting in increased recruitment of cytotoxic T lymphocytes. A pilot clinical study in advanced colon cancer patients treated with rituximab reported encouraging tumor regressions [34]. Therefore it is possible that depletion of B cells with rituximab also played a role in the response of HNSCC to R-CHOP in this case. It should be noted that in addition to B cell depletion from rituximab, the treatment of lymphoma with R-CHOP possibly led to broad immunologic changes, restoring immune function in general.

The excellent response could also be attributed to the inherent treatment-sensitive biology of HPV^+^ SCC. HPV^+^ patients have better progression-free survival, lower locoregional failure rates, and improved 3-year overall survival in the setting of treatment with sequential chemoradiation and even after radiotherapy alone [35]. Therefore HPV positivity may confer a more favorable prognosis in a “platform-independent” manner. In a prospective trial of low-risk HPV^+^ patients (T1-3 N0-N2b), 3 cycles of induction chemotherapy with cisplatin, paclitaxel, and cetuximab achieved an excellent complete clinical response (cCR) rate of 70%, which subsequently enabled them to be treated with a substantially lower dose of radiation (54Gy vs. 69.3Gy) [36]. In fact, the benefit of chemotherapy is unclear in this selected group of low-risk patients and efforts are underway to evaluate if chemotherapy can be omitted altogether ([35] and clinical trial NCT02254278).

In the present case, we would not have been able to observe the therapeutic effect of R-CHOP on SCC had we decided to treat SCC first with concurrent CRT. The main reason we prioritized treating lymphoma was the concern that the index retroperitoneal mass represented transformed lymphoma. However, this could not be ascertained without a tissue biopsy which was contraindicated due to the large size and vascularity of the mass. In addition, the high proliferation rate of the surrounding nodal disease, bulky disease, and relatively good prognosis of SCC all conspired for an upfront aggressive chemotherapy regimen like R-CHOP.

## Conclusions

We report a case of synchronous retroperitoneal follicular lymphoma and HPV^+^ BOT HNSCC in a 64 year-old female patient in which HNSCC had an excellent response to R-CHOP chemotherapy before definitive chemoradiation therapy. Although there are published case reports of synchronous SCC of the aerodigestive tract and NHL [22–24, 37–48], this is the first to report a dramatic response of SCC to R-CHOP.This case highlights the exquisite sensitivity of HPV-related HNSCC, which should be taken into consideration in treatment prioritization in the setting of a concurrent diagnosis of a second cancer. The exact agent(s) responsible for the observed response is unclear but the immunomodulatory effect of rituximab and/or the cytotoxic effect of cyclophosphamide, doxorubicin and vincristine could each have played a role.

## Abbreviations

5-FU: 5-fluorouracil
BOT: Base of tongue
CABO: Cisplatin, methotrexate, bleomycin and vincristine
cCR: Complete clinical response
CRT: Concurrent chemoradiation
CT: Computed tomography
EGFR: Epidermal growth factor receptor
HNSCC: Squamous cell carcinoma of the head and neck
HPV: Human papillomavirus
NSTEMI: non-ST-elevation myocardial infarction
OPC: Oropharyngeal cancer
PET/CT: Positron emission tomography/computed tomography
R-CHOP: Rituximab, cyclophosphamide, doxorubicin, vincristine, and prednisone
RT: Radiation therapy
SCC: Squamous cell carcinoma
SPC: Second primary cancer
